# Causative Agent of Pogosta Disease Isolated from Blood and Skin Lesions

**DOI:** 10.3201/eid1005.030689

**Published:** 2004-05

**Authors:** Satu Kurkela, Tytti Manni, Antti Vaheri, Olli Vapalahti

**Affiliations:** * Haartman Institute, Helsinki, Finland; † HULAB, Helsinki, Finland; ‡ University of Helsinki, Helsinki, Finland

**Keywords:** Alphavirus, Arboviruses, Exanthema, Sindbis virus, Viral arthritis, Viremia, Zoonoses

## Abstract

Pogosta disease is a mosquito-borne viral disease in Finland, which is clinically manifested by rash and arthritis; larger outbreaks occur in 7-year intervals. The causative agent of the disease has been suspected of being closely related to Sindbis virus (SINV). We isolated SINV from five patients with acute Pogosta disease during an outbreak in fall 2002 in Finland. One virus strain was recovered from a whole blood sample and four other strains from skin lesions. The etiology of Pogosta disease was confirmed by these first Finnish SINV strains, which also represent the first human SINV isolates from Europe. Phylogenetic analysis indicates that the Finnish SINV strains are closely related to the viral agents isolated from mosquitoes and that cause clinically similar diseases in nearby geographic areas.

Sindbis virus (SINV), a member of the Western equine encephalomyelitis virus complex of the genus *Alphavirus* in the family *Togaviridae*, was first isolated in 1952 in the Nile River delta in Egypt from a pool of *Culex pipiens* and *Cx*. *univittatus* mosquitoes (*1*). SINV is an enveloped virus with a genome of single-stranded, positive-polarity 11.7-kb RNA (*2*). The genomic 49S RNA also serves as mRNA in the infected cell. Translation of the genomic RNA produces the four nonstructural proteins nsP1–4. The 26S subgenomic mRNA is translated to produce the polyprotein from which E1, E2, and C structural proteins are processed.

The seroprevalence of SINV antibodies among the Finnish population is approximately 2% (*3*); however, the prevalence varies considerably between different parts of the country. The typical clinical picture of Pogosta disease includes arthritis, maculopapular rash, and sometimes low fever, fatigue, and muscle pain (*4*; Kurkela et al., unpub. data). Clinically similar or identical diseases are Ockelbo disease (known as August–September disease) in Sweden and Karelian fever in Russian Karelia (*5*–*7*). Ockelbo disease was first found in Sweden in 1967 (*8*). A larger Pogosta disease outbreak has occurred every 7th year in Finland since 1974, with hundreds or thousands of patients (*3*). The 7-year cycle occurred again in August through September 2002 as well, with 597 serodiagnosed cases reported. The area of highest incidence (8.0/10 000 person-years) was, as in previous epidemic years, the province of North Karelia in eastern Finland, with an incidence almost eight times higher than in the whole country in general. The cause for this geographic distribution is not known. In Sweden, the seroprevalence of SINV antibodies has been highest in the central parts of the country (*9*). Since all the clinical cases in Finland occur in late July through early October, the virus is most likely carried and transferred to humans by the late summer mosquito species *Culex* and *Culiseta*, from which SINV has previously been isolated in Sweden (*10*). Possible viral reservoirs of SINV are tetraonid and migratory birds, which are a major blood source for mosquitoes and have been shown to harbor SINV antibodies (*3*,*11*,*12*).

SINV has been isolated from various insects and vertebrates around the world (Figure 1). From a human sample, however, SINV isolation has previously been documented twice. South African Girdwood strain was recovered in 1963 from the vesicle fluid of skin lesions taken from a 45-year-old woman with acute rash-arthritis (*13*), and Chinese YN87448 strain was isolated from the serum of a febrile patient in 1992 (*14*). Viral RNA has been detected from skin lesions with the polymerase chain reaction (PCR) method in Sweden (*15*), but no genetic sequence is available. No virus isolations or detections have previously taken place in Finland. The aim of this study was to isolate the causative agent of Pogosta disease directly from human samples.

**Figure 1:**
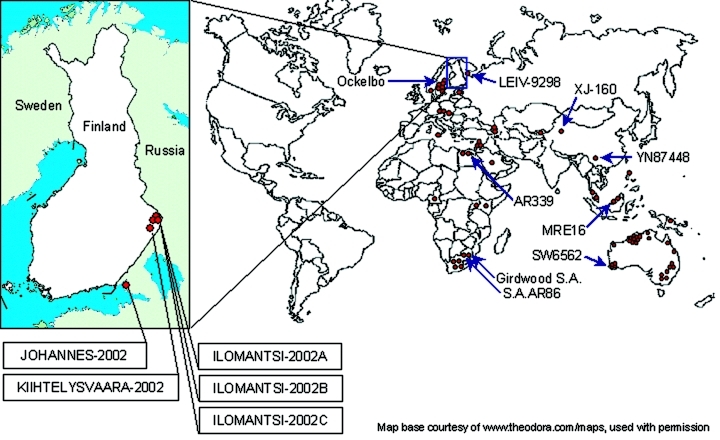
Sindbis virus isolates around the world. Each dot represents either one strain (isolated from insect or vertebrate) or several strains isolated from specific *Diptera* genus (e.g., *Culex* or *Aedes*) at the same time and place. The strains included in the phylogenetic analyses are indicated with arrows. The enlarged map presents the new Sindbis virus isolates introduced in this study.

## Patients and Methods

### Clinical Samples

We collected samples from the health districts where the incidence of Pogosta disease had been highest during previous epidemic years, North Karelia and Kuopio. When acute SINV infection was suspected on clinical examination, whole blood samples in tubes containing ethane diamine tetraacetic acid (EDTA) as anticoagulant and skin lesion biopsy specimens were collected, if possible. The skin biopsy specimens were taken from one papulopustule with a punch or a surgical knife into a dry tube, usually by a local physician. The samples were kept at +4°C, and transported to the local central hospital usually within the next 24 hours to be frozen at –70°C. All samples were kept frozen until they were processed at the department of Virology, University of Helsinki. Informed consent was obtained from all patients. The study was carried out under the permission of the coordinating ethical committee of the Hospital District of Helsinki and Uusimaa.

### Representative Case Report

A 35-year-old man was most probably exposed to SINV in August 2002, in Ilomantsi, a province of North Karelia. Typical Pogosta disease symptoms began with polyarthritis, first affecting the fingers, wrists, and the left shoulder, then extending to the left elbow, and finally both knees and the right ankle. Joint symptoms were often aggravated by exertion. At the acute phase, the patient also had fatigue, nausea, muscle pain, headache, low fever, and itchy rash throughout the body. A skin biopsy was taken from one papulopustule (see also Table; patient 1). Basic blood parameters at the acute phase were within normal range, including blood cell counts, hemoglobin, platelets, and the erythrocyte count indices. The joint symptoms lasted for 3 to 4 months. Besides these symptoms, no other residual symptoms were encountered.

### Serodiagnostic Methods

All patients were screened for SINV immunoglobulin (Ig) M and IgG with enzyme immunoassay (EIA) and for total antibodies with the hemagglutination inhibition test (HI). EIA was performed with purified SINV antigen, directly coated on microtiter well plates (*16*). For HI microtitration, serum samples were absorbed with kaolin and male goose erythrocytes and tested against SINV (grown in BHK21/WI-2 cell monolayers) and 0.2% suspension of goose erythrocytes (*3*). For the diagnosis, either a positive result in EIA for SINV IgM in a single sample or seroconversion (or greater than fourfold increase in titer) between paired serum specimens was required.

### Virus Isolation

The frozen skin samples were cut into small pieces, then homogenized in a mortar, and suspended in 100–150 μL Dulbecco’s minimal essential medium plus 0.2% bovine serum albumin. A total of 50–100 μL of this suspension diluted in 500 μL of culture medium, containing minimal essential medium and 2% fetal calf serum with a mixture of glutamine, ampicillin, and penicillin, was added to confluent Vero cells in 25-cm^2^ cell culture flasks. Whole blood samples were diluted 1:10 in culture medium, and confluent Vero cell cultures were injected with the final volume of 500 μL. The cells were incubated for 1 h at 37°C, then 3–4 mL culture medium was added, and the cultures were kept at 37°C. The toxicity of EDTA-anticoagulant on Vero cells could be avoided by completely removing the blood dilution from cells after the 1-h incubation and also by changing the culture medium the day after the injection and then twice a week. All cell cultures were inspected daily. When cytopathic effect (CPE) was apparent, immunofluorescence assay (IFA) was performed, and the cells were passaged.

Several measures were undertaken to avoid laboratory contamination. SINV was not handled in the two laboratories where the samples were prepared and the cells were cultured, and separate sets of instruments were always used. Samples were handled and cells cultured in separate laboratories. Cross-contamination was avoided by culturing in cell culture flasks instead of culture plates. At least two flasks of noninfected Vero cells were cultured in the same incubator every time and went through the same procedures as the potentially infected cells.

### IFA

An IFA was developed to confirm SINV infection of the cells. Cells were harvested in 600 μL phosphate-buffered saline (PBS), washed, and centrifuged at 1,800 rpm for 3 min five times and dried on a slide. For immunofluorescence staining the slides were fixed for 7 min in ice-cold acetone. A pool of 10 SINV IgG–positive serum samples were diluted 1:20 in PBS, added to slides, and incubated in a moist chamber at 37°C for 30 min. The slides were washed three times in PBS and once in water, then incubated at 37°C for 30 min with fluorescein isothiocyanate–conjugated F(ab')2 goat anti-human IgG diluted 1:100 in PBS. After another wash, the slides were dried, mounted, and screened with a fluorescence microscope.

### RNA Extraction and Reverse Transcription (RT)-PCR

Viral RNA was extracted from culture supernatant with TriPure Isolation Reagent (Roche Diagnostics [Roche Molecular Biochemicals, Espoo, Finland]) by following the manufacturer’s instructions. The primers used in RT-PCR were 5′-tttagcggatcggacaattc-3′ and 5′-gcggtgacgaactcagtag-3′. The RT reaction was carried out as follows: 10 μL of RNA dissolved in water was mixed with 2 μL of each primer (10 pmol/μL), 2 μL of M-MuLV reverse transcriptase (20 U/μL [MBI Fermentas, Vilnius, Lithuania]), 5 μL of 5 x RT-buffer (MBI Fermentas), 2 μL of dNTP-mix (2.5mM dATP, dTTP, dGTP, and dCTP [Finnzymes, Espoo, Finland]), and 2 μL of Ribonuclease inhibitor (40 U/μL; MBI Fermentas). The mixture was incubated at 37°C for 90 min.

For the PCR 7 μL of cDNA were incubated at 95°C for 5 min, cooled immediately on ice, and mixed with 2 μL of each primer (10 pmol/μL), 10 μL of 10 x Taq Extender PCR Buffer (Stratagene, La Jolla, CA), 8 μL of dNTP mix (2.5 mM; Finnzymes), 1 μL of Taq DNA Polymerase (recombinant) (5 U/μL; MBI Fermentas), 1 μL of Taq Extender PCR Additive (Stratagene), and 69 μL of water. The reactions were amplified through 35 cycles using a DNA thermal cycler with the following steps: denaturation at 95°C for 45 s, annealing at 55°C for 2 min, and elongation at 72°C for 3 min, followed by a final extension at 72°C for 10 min.

### Cloning

The PCR amplicons were purified with QIAquick gel extraction kit (Qiagen, Hilden, Germany). The amplicons were cloned with TOPO TA Cloning Kit for Sequencing (Invitrogen, Carlsbad, CA), following the manufacturer’s instructions, and transformed into TOP10 chemically competent *Escherichia coli* cells on bacterial plates containing x-gal (5-bromo-4-chloro-3-indolyl-β-D-galactoside) and IPTG (isopropylthio-β-D-galactoside) for blue-white screening. The plasmid DNA was isolated with QIAprep Miniprep kit (Qiagen). and restriction analysis was performed. Vector-based primers M13 Reverse and T7 were used for automatic sequencing with ABI PRISM (Perkin-Elmer, Foster City, CA).

### Sequence Analysis and Phylogenetic Analysis

Sequences were aligned with Clustal W1.75 program (*17*) into MSF-format and edited with GeneDoc Multiple Sequence Alignment (available from: http://www.psc.edu/biomed/genedoc/) editor program. Sequence was confirmed from at least three different clones. PHYLIP program package (Felsenstein, 1993) was used to create 5,000 bootstrap replicates on the sequence data (SEQBOOT). Distance matrices were calculated with DNADIST program with Kimura’s two-parameter model of substitutions and analyzed by Neighbor-joining tree-fitting algorithm with NEIGHBOR program. The bootstrap support values were calculated with CONSENSE program.

## Results

Altogether 131 patients with suspected acute Pogosta disease were recruited to the study in 11 different health stations in the province of North Karelia and in Kuopio University Hospital, Finland, during July through October of 2002. A total of 86 patients had acute, serologically confirmed SINV infection. Twenty-three skin biopsy specimens and 73 whole-blood samples (treated with EDTA) were collected from patients with acute SINV infection and used in this study for isolation attempts.

A physician examined all patients at the acute phase. To determine the most likely place and time of exposure to SINV, background information about the physical location of the patients before the onset of symptoms was also collected by using questionnaires. The exact incubation period in Pogosta disease is unknown, and determining the time and place of exposure was difficult.

Virus isolation was successful from 4 of 23 skin samples and 1 of 73 whole blood samples (Table) (Figure 1). All isolates induced a strong CPE in cell culture, featuring round-shaped, interconnected cells and cell death within 72 hours of culturing. Infected cells from all isolates were shown to give a strong positive signal with IFA with human SINV IgG–positive sera. All noncytopathic cell cultures were also screened and found negative with IFA, e.g., no discrepancy between the isolation method and immunofluorescence staining was encountered.

**Table:** Samples from which Sindbis virus (SINV) was isolated^a,b^

Patient	Strain	Sample	Sex	Age (y)	Probable place of exposure	Onset of symptoms	Disease/rash days at time of sampling	SINV IgM status at time of sampling	
1	Ilomantsi-2002A	Skin lesion	M	35	Ilomantsi, Finland	8/ 22/2002	3/ 1	Negative	
2	Ilomantsi-2002B	Skin lesion	M	30	Ilomantsi, Finland	8/18/2002	2/ 0	Negative	
3	Ilomantsi-2002C	Whole blood	F	47	Ilomantsi, Finland	Late Aug. 2002	N/A	Borderline	
4	Johannes-2002	Skin lesion	M	63	Johannes, Russia	8/27/2002	9/7	N/A	
5	Kiihtelysvaara-2002	Skin lesion	M	39	Kiihtelysvaara, Finland	8/30/2002	3/ 2	Negative	

^a^The places of exposure are shown on the map (Figure 1). The representative case report given in the text describes the patient from whom the Ilomantsi-2002A virus strain was recovered. ^b^M, male; F, female; N/A, not available; Ig, immunoglobulin.

The median and average number of days of rash for patients with the positive skin samples before sampling were 1.5 and 2.5, respectively, and SINV IgM antibodies were not detectable (IgM status for patient 4 at the time of sampling was not available) (Table). The median and average days of rash for the negative skin samples until sampling were 2.0 and 1.7, respectively (data available from 17 of 19 patients). In all, 37% of the patients who gave a skin biopsy sample had detectable SINV IgM antibodies: 16% had a borderline result at the time of sampling (data available from 19 of 23 patients). The median and average number of disease days before collecting the whole blood samples were 3.0 and 4.5, respectively (data available from 65 of 73 patients). The only blood sample from which SINV could be isolated had a borderline SINV IgM result; the exact time of onset of symptoms is not available for this case.

**Figure 2:**
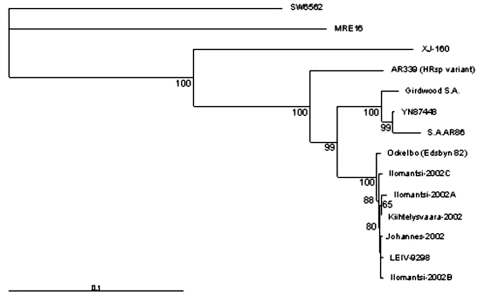
Phylogenetic tree is based on the nucleotide sequences of 1,178–1,281 bp from nsP3 and nsP4 region, nucleotides 5,258-6,510; the genome position is given according to the published sequence of the strain AR339 (HRsp variant) (*2*). The tree was constructed by using Neighbor-joining algorithms (NEIGHBOR). 5,000 bootstrap replicates were calculated. Only those bootstrap support values that exceed 50% are shown. The following sequences available in GenBank were included into the comparison: AR339 (HRsp variant); Egypt (J02363, J02364, J02365, J02366, J02367), Girdwood S.A.; South-Africa (U38304), MRE16; Malaysia (AF492770), Ockelbo (Edsbyn 82); Sweden (M69205), S.A.AR86; South-Africa (U38305), SW6562; Australia (AF429428), YN87448; China (AF103734) and XJ-160; China (AF103728).

The nucleotide sequences of 1,178–1,281 bp from nsP3 and nsP4 region of the new strains were determined for the phylogenetic analysis and submitted to GenBank. The strains were given the following accession numbers: Ilomantsi-2002A (AY532322), Ilomantsi-2002B (AY532326), Ilomantsi-2002C (AY532324), Kiihtelysvaara-2002 (AY532325), and Johannes-2002 (AY532323). In addition, we sequenced from this region the LEIV-9298-strain (AY532321), isolated from *Aedes* mosquitoes in 1983 in central Russian Karelia, approximately 200 km north of Ilomantsi, Finland (*6*). The following sequences available in GenBank were included in the comparison: AR339 (HRsp variant), Girdwood S.A., MRE16, Ockelbo (Edsbyn 82), S.A.AR86, SW6562, YN87448, and XJ-160. See Figure 1 for the geographic location and Figure 2 for the phylogenetic tree of the strains. Sequence comparisons and phylogenetic analysis show that the northern European (e.g, Finnish, Russian, and Swedish) SINV strains analyzed in this study are closely related, with a percentage difference of 0.1% to 1.4% on nucleotides and 0% to 2.1% on amino acids. The Russian Karelian LEIV-9298 and Johannes differ by one nucleic acid, and their amino acid sequences are identical. Malaysian MRE16 is furthest from Finnish strains when both nucleic and amino acids are compared, differences are 35.6% to 35.8% and 28.3% to 28.5% difference, respectively.

## Discussion

This study describes the first human SINV isolates from Europe, one strain from blood and four from skin lesions. One of the strains (Johannes-2002) is apparently Russian, since the Finnish patient most likely was exposed to SINV in Russian Karelia. The four other strains represent the first SINV isolates from Finland. Phylogenetic analysis of the strains shows a close relationship to Swedish and Russian SINV strains, isolated approximately 20 years ago from mosquitoes.

The possibility of laboratory contamination was minimized by various measures as described in Methods. Only a few samples were prepared at the same time; each new virus strain was isolated from separate set of samples. No CPE was apparent in the negative control cells at any stage and they were all negative in immunofluorescence staining as well.

SINV could be recovered from one blood sample of 73. This sample was positive by nested RT-PCR method as well; full characterization of clinical and laboratory data will be presented later (Kurkela et al., unpub. data). As now proven, SINV is present in the blood during acute infection. The most viremic window appears to be very narrow and the level of viremia can vary considerably between persons. These presumptions make future laboratory diagnostics based on viral detection challenging, and serology likely will remains the method of choice for diagnosis.

In skin tissue, the viral persistence seems to last for several days, if not weeks. The Johannes-2002 strain was isolated from a biopsy specimen taken 7 days after the onset of rash. SINV could be recovered from 17% of the skin biopsy samples. Whether the virus persists in synovial fluid and whether this could explain the prolonged joint symptoms in a substantial proportion of Pogosta disease patients (Kurkela et al., unpub. data) remain to be determined. Some patients have had borderline results in IgM serologic testing even months or years after the onset of disease (*18*), although no correlation between prolonged joint symptoms and elevated IgM levels in serum has been observed. In experiments with mice, evidence indicates that viral replication can take place in the periosteum or endosteum (*19*). Efforts to detect SINV or SINV RNA from human synovial fluids have failed (*20*). Clinical indications to retrieve synovial fluid samples rarely occur in Pogosta disease, and therefore no synovial fluids were available in this study.

The cell type in which the virus replicates in skin tissue is not known. In histopathologic examination of skin lesions of Pogosta disease patients, a pronounced lymphohistiocytic inflammatory infiltrate and lymphoblast-like cells have been described (*21*). Since the virus is present in the skin during acute infection before the onset of antibody response (Table), the cutaneous manifestations in Pogosta disease may be due to a direct viral effect; however, a more complex immunologic reaction behind the pathogenesis can certainly not be excluded.

Four patients were serodiagnosed with acute SINV infection in Sweden during the Finnish outbreak in 2002 (Sirkka Vene, pers. comm.), which suggests that local ecologic and environmental factors are involved in the epidemiology of Pogosta disease in Finland. Data are not available about whether the epidemics in northern Karelia were accompanied by similar epidemics on the Russian side of the Finnish-Russian border, although one of our patients (Table; patient 4) was most probably exposed while traveling in Russian Karelia. Ecologic circumstances likely alter the viral cycle in nature, and the prevalence of mosquitoes carrying SINV varies. However, differences in the pathogenicity of viral strains cannot be excluded. In Australia, for instance, SINV is the most common arbovirus isolated from mosquitoes (*22*), but human infections have often been either subclinical or mild (*23*–*25*). Furthermore, the virus has been isolated from different animals have also taken place in central Europe without any reported human cases.

Phylogenetic analysis of the Finnish SINV strains for a 1.2-kb genome segment indicates that Finnish SINV strains are closely related to each other and to the Swedish and Russian strains, isolated 2 decades ago (Figure 2). Within this genome segment, Johannes-2002 strain is almost identical to LEIV-9298 strain, isolated 20 years earlier, ≈500 km north of Johannes. Northern European SINV strains might constitute a restricted geographic area of viral emergence, which suggests that the virus is maintained locally in disease-endemic regions. However, viral redistribution over long distances is possible, which would introduce new, distinct SINV strains to northern Europe. Viral recombination could also account for further variation, but more sequence information from other gene regions and SINV strains is required to define its role.

South African SINV strains have been shown to not vary substantially from Swedish strains (*26*). Therefore, the question still remains how and from where the viral importation to Finland has taken place. One possibility could be through mosquitoes carried by air currents. Migratory birds would be plausible viral carriers from further distances, such as Africa. Up to 50% of migratory and tetraonid birds have been shown to carry SINV antibodies in Finland (*3*); birds could function both as viral importers and amplifying hosts (*11*,*12*). Resident and migratory birds have recently been shown to carry SINV antibodies also in the United Kindom (*26*). The phylogenetic analysis in this study supports this concept by suggesting that redistribution of SINV tends to occur in a longitudinal, not latitudinal, direction. Contrary to the above, the strain YN87448, which originates from eastern China, is positioned in the phylogenetic tree close to the African strains (Figure 1). Indications of periodic redistribution of SINV strains over long distances and within a short time have previously been demonstrated in Australia (*28*), a finding consistent with the involvement of migratory birds. SINV could be frequently introduced to Finland or hide locally through winter in some natural reservoir, to emerge again in August through September. The available data favor maintenance of SINV in a local endemic cycle but the extent to which new introductions of SINV may play a role remains a subject for further investigation.
